# Supplementary figures and images for: A stacked ensemble method for forecasting influenza-like illness visit volumes at emergency departments
Source: PLoS One. 2021 Mar 22;16(3):e0241725. doi: 10.1371/journal.pone.0241725 (PMC7984626; doi:10.1371/journal.pone.0241725)

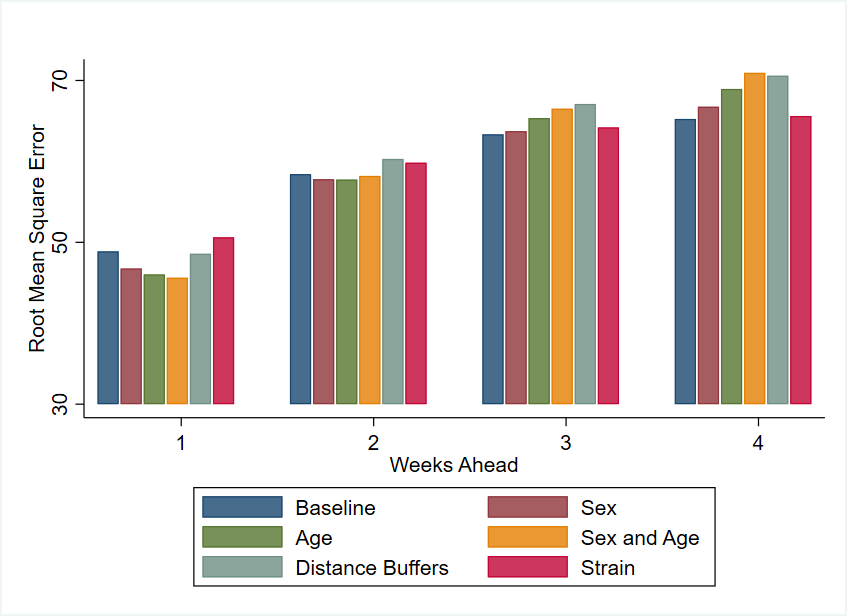

Supplement: S1 Fig — Notes. (1) Data from weekly ILI visits at the ACH. (2) The Baseline QRF is described in section 2.2; the other QRF specifications build on the Baseline. E.g., Sex means we disaggregate weekly ILI counts by male and female patients. (3) Calculation of RMSE uses 42 weeks on each test season of the ACH data, starting from epiweek 37 or approximately mid September. (4) Due to data availability, the 2018 season uses only 14 weeks of data, also starting from epiweek 37. (TIF) [file pone.0241725.s001.tif]

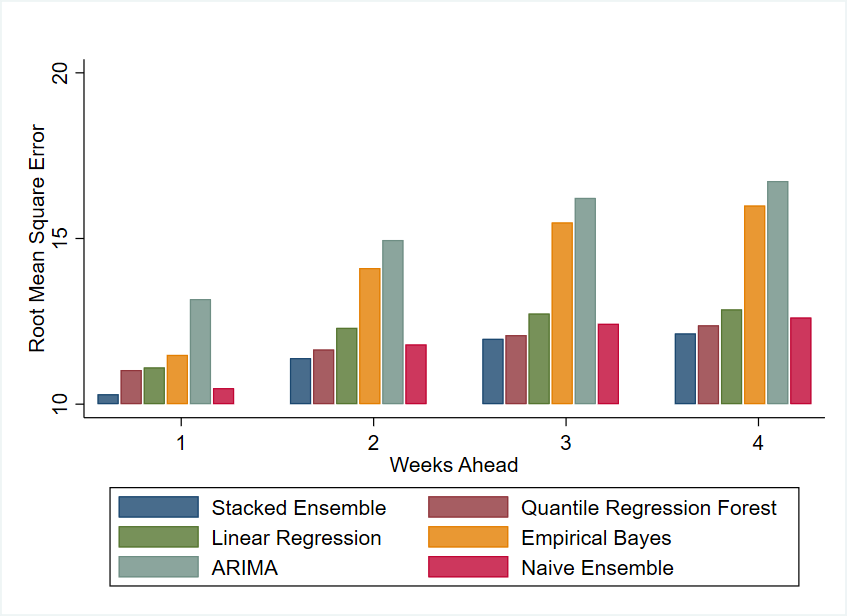

Supplement: S2 Fig — (TIF) [file pone.0241725.s002.tif]
