# Supplementary material for: A stacked ensemble method for forecasting influenza-like illness visit volumes at emergency departments
Source: PLoS One. 2021 Mar 22;16(3):e0241725. doi: 10.1371/journal.pone.0241725 (PMC7984626; doi:10.1371/journal.pone.0241725)
Supplement: S1 Data — (ZIP) [file pone.0241725.s003.zip › ILI_plosone/readme.pdf]

Reproduction readme file

A stacked ensemble method for forecasting influenza-like illness visit volumes at emergency departments

by: Arthur Novaes de Amorim

Last updated: January 22, 2021

1 Introduction

This readme file provides an overview of the reproduction data and codes for "A stacked ensemble method for forecasting influenza-like illness visit volumes at emergency departments."

2 Directory Tree

Unzipping the reproduction data and codes zipfile yields the following directory structure

```
Root/
├── data/
│   └── clean/
│       └── minimal_reproduction_data.xlsx
├── programs/
│   ├── 0.dir_setup.do
│   └── 1.tables_figures.do
└── tables/
    └── *.csv
```

The clean data folder contains the minimal data set for generating our results and findings. Running the models, as described in the manuscript, yields the performance metrics stored in various csv files in the tables directory. These are converted into the tables and figures shown in the paper by running the two Stata programs located in the programs directory.

For reproducing the figures and tables, please first open the do file `0.dir_setup.do`, located under the programs directory, and insert the path to the project root folder at line 17 of the do file. Run this do file, and then run the do file `1.tables_figures.do`, also located in the programs directory, to generate the tables and figures from the paper.

A note: model names have been revised during the preparation of this project. Some names displayed in the output csv files are outdated. Where applicable, “Random Forest” refers to “Quantile Regression Forest,” “Model Average” refers to “Stacked Ensemble,” and “Naive Average” refers to the “Naive Ensemble” in the tables and figures in the paper. These naming conventions are corrected during the execution of the do file `1.tables_figures.do`.
